# Supplementary material for: Minimum dataset with integrated scoring and indexing methods for soil quality assessment
Source: PLoS One. 2026 Apr 7;21(4):e0346136. doi: 10.1371/journal.pone.0346136 (PMC13056203; doi:10.1371/journal.pone.0346136)
Supplement: S1 Table — (DOCX) [file pone.0346136.s001.docx]

**S1 Table.** Control treatment corn yield in Alabama, Indiana, and Ohio (Hoytville and Piketon) from 2012 to 2016 (average of four replications).

| Corn yield (bu./ac)  **Indiana** | Corn yield (bu./ac)  **Hoytville** | Corn yield (bu./ac)  **Alabama** | Corn yield (bu./ac)  **Piketon** |
| --- | --- | --- | --- |
| (2012-2016) | (2012-2016) | (2012-2016) | (2012-2016) |
| 134 | 192 | 144 | 95 |
| 130 | 191 | 114 | 69 |
| 153 | 204 | 184 | 104 |
| 171 | 202 | 136 | 109 |
| 169 | 169 | 117 | 104 |
| 177 | 191 | 128 | 119 |
| 146 | 169 | 135 | 75 |
| 200 | 172 | 117 | 27 |
| 163 | 150 | 113 | 180 |
| 183 | 158 | 72 | 131 |
| 167 | 146 | 110 | 157 |
| 177 | 129 | 83 | 177 |
| 231 | 156 | 25 | 155 |
| 224 | 158 | 103 | 139 |
| 221 | 147 | 65 | 140 |
| 234 | 143 | 26 | 121 |

Control treatment: The control treatment is defined as conventional soybean–corn rotation under no-till management, with no gypsum application (0 Mg/ha) and no cover crop.
