# Supplementary material for: Minimum dataset with integrated scoring and indexing methods for soil quality assessment
Source: PLoS One. 2026 Apr 7;21(4):e0346136. doi: 10.1371/journal.pone.0346136 (PMC13056203; doi:10.1371/journal.pone.0346136)
Supplement: S2 Table — (DOCX) [file pone.0346136.s002.docx]

**S2 Table.** Descriptive statistics of controlled treatment soil properties at Indiana site in 2012-2016 (average of 4 replications).

| Soil properties | Mean | Std | SEM | Skewness | Kurtosis | CV | Min | Median | Max |
| --- | --- | --- | --- | --- | --- | --- | --- | --- | --- |
| SMB (mg/kg) | 219 | 130 | 26.6 | 1.53 | 2.13 | 0.59 | 55.8 | 181.50 | 589 |
| Non-SBM (%) | 1.26 | 0.39 | 0.08 | -0.21 | -0.52 | 0.31 | 0.46 | 1.25 | 1.91 |
| qR (%) | 1.85 | 1.18 | 0.24 | 1.23 | 0.70 | 0.64 | 0.55 | 1.32 | 4.67 |
| pH | 6.55 | 0.50 | 0.10 | 0.32 | 0.27 | 0.08 | 5.50 | 6.51 | 7.67 |
| ECe (µS/cm) | 183. | 33.51 | 6.84 | 0.38 | -0.57 | 0.18 | 135 | 179 | 258 |
| TN (%) | 0.14 | 0.04 | 0.01 | -0.21 | -0.70 | 0.26 | 0.07 | 0.14 | 0.20 |
| SOC (%) | 1.28 | 0.39 | 0.08 | -0.24 | -0.48 | 0.31 | 0.47 | 1.29 | 1.94 |
| AC (mg/kg) | 5321 | 154 | 31.4 | -0.61 | -0.33 | 0.29 | 188 | 535.90 | 746.6 |
| NPI | 1.41 | 0.34 | 0.07 | 0.37 | -0.41 | 0.24 | 0.83 | 1.34 | 2.19 |
| CPI | 1.22 | 0.34 | 0.07 | -0.26 | -0.91 | 0.28 | 0.51 | 1.16 | 1.71 |
| CL | 0.04 | 0.01 | 0.00 | -0.09 | 0.03 | 0.17 | 0.03 | 0.04 | 0.06 |
| Cli | 1.26 | 0.22 | 0.04 | -0.09 | 0.03 | 0.17 | 0.74 | 1.24 | 1.67 |
| CMI | 1.50 | 0.36 | 0.07 | -1.06 | 0.44 | 0.24 | 0.61 | 1.60 | 1.92 |
| nCMI | 69.3 | 16.40 | 3.35 | -1.06 | 0.44 | 0.24 | 28.0 | 73.7 | 88.5 |
| pb (g/cm^3^) | 1.33 | 0.12 | 0.02 | 0.00 | -0.73 | 0.09 | 1.14 | 1.35 | 1.55 |
| MaAS (%) | 59.1 | 3.68 | 0.75 | -0.41 | -0.44 | 0.06 | 52.21 | 59.81 | 65.7 |
| MiAS (%) | 7.30 | 2.47 | 0.50 | -0.26 | 0.12 | 0.34 | 1.24 | 6.91 | 11.9 |
| AS (%) | 66.4 | 3.26 | 0.67 | -0.88 | 0.51 | 0.05 | 58.3 | 66.8 | 70.3 |
| SI | 10.3 | 9.48 | 1.93 | 4.29 | 19.8 | 0.92 | 4.59 | 8.18 | 53.0 |
| PI | 13.4 | 3.99 | 0.82 | 0.70 | 1.01 | 0.30 | 5.03 | 12.9 | 23.4 |
| MWD (mm) | 1.16 | 0.41 | 0.08 | 0.92 | 0.60 | 0.36 | 0.40 | 1.08 | 2.09 |
| GMD (mm) | 0.96 | 0.28 | 0.06 | 1.33 | 2.06 | 0.29 | 0.51 | 0.92 | 1.75 |

SMB: soil microbial biomass; Non-SMB: non-microbial biomass carbon; qR: microbial biomass carbon over total organic carbon; ECe: electric conductivity of soil; TN: total nitrogen; SOC: total carbon; AC: active carbon; NPI: nitrogen pool index; CPI: carbon pool index; CL: carbon lability; Cli: carbon lability index; CMI: carbon management index; nCMI: normalized carbon management index; pb: soil bulk density; MaAS: macroaggregate stability; MiAS: microaggregate stability; AS: total aggregate stability; SI: stability index; and PI: persistent index, MWD: Mean weight diameter; GMD: Geometric mean diameter. Control treatment:

The control treatment is defined as conventional soybean–corn rotation under no-till management, with no gypsum application (0 Mg/ha) and no cover crop.
