# Supplementary material for: Minimum dataset with integrated scoring and indexing methods for soil quality assessment
Source: PLoS One. 2026 Apr 7;21(4):e0346136. doi: 10.1371/journal.pone.0346136 (PMC13056203; doi:10.1371/journal.pone.0346136)
Supplement: S3 Table — (DOCX) [file pone.0346136.s003.docx]

**S3 Table.** Descriptive statistics of controlled treatment soil properties at Hoytville (Ohio) site (average of 4 replications).

| Soil properties | Mean | Std | SEM | Skewness | Kurtosis | CV | Min | Median | Max |
| --- | --- | --- | --- | --- | --- | --- | --- | --- | --- |
| SMB (mg/kg) | 333 | 126 | 26.2 | 0.76 | 1.09 | 0.38 | 150.22 | 322 | 680 |
| Non-SMB (%) | 1.88 | 0.12 | 0.02 | 0.76 | 2.73 | 0.06 | 1.61 | 1.86 | 2.23 |
| qR (%) | 1.73 | 0.62 | 0.13 | 0.98 | 1.67 | 0.36 | 0.84 | 1.68 | 3.51 |
| pH | 6.00 | 0.57 | 0.12 | 1.82 | 5.99 | 0.10 | 5.20 | 5.94 | 7.99 |
| ECe (µS/cm) | 291 | 140 | 29.1 | 1.85 | 3.01 | 0.48 | 157 | 223 | 713 |
| Total N (%) | 0.20 | 0.01 | 0.00 | 0.14 | 1.30 | 0.05 | 0.18 | 0.20 | 0.22 |
| SOC (%) | 1.92 | 0.13 | 0.03 | 0.60 | 2.51 | 0.07 | 1.63 | 1.91 | 2.27 |
| AC (mg/kg) | 463 | 91.7 | 19.1 | -0.48 | -0.67 | 0.20 | 255 | 465 | 581 |
| NPI | 0.98 | 0.04 | 0.01 | -0.25 | -0.78 | 0.05 | 0.89 | 0.98 | 1.04 |
| CPI | 1.02 | 0.06 | 0.01 | 0.45 | 2.02 | 0.05 | 0.90 | 1.02 | 1.18 |
| CL | 0.02 | 0.00 | 0.00 | -0.63 | 0.19 | 0.17 | 0.01 | 0.02 | 0.03 |
| Cli | 1.23 | 0.16 | 0.03 | -1.17 | 1.95 | 0.13 | 0.74 | 1.25 | 1.45 |
| CMI | 1.25 | 0.18 | 0.04 | -1.14 | 1.09 | 0.15 | 0.75 | 1.29 | 1.48 |
| nCMI | 64.2 | 9.42 | 1.96 | -1.14 | 1.09 | 0.15 | 38.4 | 66.3 | 75.7 |
| pb (g/cm^3^) | 1.39 | 0.06 | 0.01 | -0.08 | -0.24 | 0.05 | 1.26 | 1.39 | 1.52 |
| MaAS (%) | 55.3 | 4.05 | 0.84 | 0.10 | -0.41 | 0.07 | 48.1 | 54.8 | 63.7 |
| MiAS (%) | 11.1 | 1.90 | 0.40 | 0.20 | -0.99 | 0.17 | 8.16 | 10.9 | 14.0 |
| AS (%) | 66.4 | 3.12 | 0.65 | -0.28 | -0.30 | 0.05 | 59.5 | 66.7 | 71.9 |
| SI | 5.17 | 1.20 | 0.25 | 0.61 | -0.21 | 0.23 | 3.44 | 4.96 | 7.81 |
| PI | 19.7 | 2.94 | 0.61 | -0.45 | -0.30 | 0.15 | 13.2 | 20.0 | 24.7 |
| MWD (mm) | 1.25 | 0.19 | 0.04 | -0.07 | -1.04 | 0.15 | 0.91 | 1.22 | 1.56 |
| GMD (mm) | 0.92 | 0.10 | 0.02 | -0.19 | -0.53 | 0.11 | 0.72 | 0.90 | 1.09 |

SMB: soil microbial biomass; Non-SMB: non-microbial biomass carbon; qR: microbial biomass carbon over total organic carbon; ECe: electric conductivity of soil; TN: total nitrogen; SOC: total carbon; AC: active carbon; NPI: nitrogen pool index; CPI: carbon pool index; CL: carbon lability; Cli: carbon lability index; CMI: carbon management index; nCMI: normalized carbon management index; pb: soil bulk density; MaAS: macroaggregate stability; MiAS: microaggregate stability; AS: total aggregate stability; SI: stability index; and PI: persistent index, MWD: Mean weight diameter; GMD: Geometric mean diameter. Control treatment:

The control treatment is defined as conventional soybean–corn rotation under no-till management, with no gypsum application (0 Mg/ha) and no cover crop.
