# Supplementary material for: Minimum dataset with integrated scoring and indexing methods for soil quality assessment
Source: PLoS One. 2026 Apr 7;21(4):e0346136. doi: 10.1371/journal.pone.0346136 (PMC13056203; doi:10.1371/journal.pone.0346136)
Supplement: S4 Table — (DOCX) [file pone.0346136.s004.docx]

**S4 Table.** Descriptive statistics of controlled treatment soil properties of Alabama site (average of 4 replications).

| Soil properties | Mean | Std | SEM | Skewness | Kurtosis | CV | Min | Median | Max |
| --- | --- | --- | --- | --- | --- | --- | --- | --- | --- |
| SMB (mg/kg) | 112. | 64.24 | 12.85 | 1.41 | 3.71 | 0.57 | 21.35 | 106.16 | 323.45 |
| Non-SMB (%) | 0.64 | 0.28 | 0.06 | 0.70 | 0.70 | 0.43 | 0.19 | 0.57 | 1.38 |
| qR (%) | 2.00 | 1.46 | 0.29 | 1.64 | 2.34 | 0.73 | 0.39 | 1.57 | 5.95 |
| pH | 5.66 | 0.39 | 0.08 | -0.35 | 0.09 | 0.07 | 4.70 | 5.70 | 6.31 |
| ECe (µS/cm) | 53.97 | 39.51 | 7.90 | 3.89 | 17.29 | 0.73 | 25.20 | 43.60 | 229.0 |
| Total N (%) | 0.06 | 0.03 | 0.01 | 2.08 | 6.16 | 0.46 | 0.03 | 0.05 | 0.15 |
| SOC (%) | 0.65 | 0.28 | 0.06 | 0.75 | 0.82 | 0.43 | 0.20 | 0.58 | 1.41 |
| AC (mg/kg) | 188. | 95.66 | 19.13 | 1.42 | 5.07 | 0.51 | 14.69 | 181.00 | 517.1 |
| NPI | 1.79 | 0.48 | 0.10 | 0.91 | 0.34 | 0.27 | 1.11 | 1.62 | 3.06 |
| CPI | 1.31 | 0.35 | 0.07 | -0.66 | -0.02 | 0.27 | 0.55 | 1.38 | 1.89 |
| CL | 0.03 | 0.01 | 0.00 | -1.33 | 2.55 | 0.33 | 0.00 | 0.03 | 0.05 |
| Cli | 0.82 | 0.26 | 0.05 | -1.54 | 3.06 | 0.32 | 0.07 | 0.82 | 1.19 |
| CMI | 1.03 | 0.38 | 0.08 | -0.51 | 0.68 | 0.37 | 0.10 | 1.06 | 1.73 |
| nCMI | 54.46 | 19.49 | 3.90 | -0.66 | 0.84 | 0.36 | 5.31 | 56.48 | 92.3 |
| pb (g/cm^3^) | 1.73 | 0.18 | 0.04 | -0.71 | 0.13 | 0.11 | 1.27 | 1.77 | 2.02 |
| MaAS (%) | 39.74 | 7.08 | 1.42 | 2.03 | 7.23 | 0.18 | 29.92 | 39.01 | 66.0 |
| MiAS (%) | 17.09 | 3.82 | 0.76 | -0.53 | 0.33 | 0.22 | 8.37 | 17.50 | 24.24 |
| AS (%) | 56.83 | 5.03 | 1.01 | 1.50 | 5.44 | 0.09 | 48.37 | 56.33 | 74.36 |
| SI | 2.58 | 1.36 | 0.27 | 2.84 | 9.74 | 0.53 | 1.45 | 2.26 | 7.89 |
| PI | 11.02 | 5.50 | 1.10 | 0.75 | -0.29 | 0.50 | 4.00 | 9.93 | 23.9 |
| MWD (mm) | 0.41 | 0.39 | 0.08 | 4.78 | 23.47 | 0.93 | 0.23 | 0.34 | 2.24 |
| GMD (mm) | 0.58 | 0.21 | 0.04 | 4.17 | 19.16 | 0.36 | 0.43 | 0.54 | 1.54 |

SMB: soil microbial biomass; NBC: non-microbial biomass carbon; qR: microbial biomass carbon over total organic carbon; ECe: electric conductivity of soil; TN: total nitrogen; SOC: total carbon; AC: active carbon; NPI: nitrogen pool index; CPI: carbon pool index; CL: carbon lability; Cli: carbon lability index; CMI: carbon management index; nCMI: normalized carbon management index; pb: soil bulk density; MaAS: macroaggregate stability; MiAS: microaggregate stability; AS: total aggregate stability; SI: stability index; and PI: persistent index, MWD: Mean weight diameter; GMD: Geometric mean diameter.

The control treatment is defined as conventional soybean–corn rotation under no-till management, with no gypsum application (0 Mg/ha) and no cover crop.
