# Supplementary material for: Minimum dataset with integrated scoring and indexing methods for soil quality assessment
Source: PLoS One. 2026 Apr 7;21(4):e0346136. doi: 10.1371/journal.pone.0346136 (PMC13056203; doi:10.1371/journal.pone.0346136)
Supplement: S5 Table — (DOCX) [file pone.0346136.s005.docx]

**S5 Table.** Descriptive statistics of controlled treatments soil properties at Piketon (Ohio) controlled soil (average of 4 replications).

| Soil properties | Mean | Std | SEM | Skewness | Kurtosis | CV | Min | Median | Max |
| --- | --- | --- | --- | --- | --- | --- | --- | --- | --- |
| SMB (mg/kg) | 0.51 | 0.20 | 0.04 | 0.42 | -1.04 | 0.40 | 0.25 | 0.54 | 0.92 |
| Non-SMB (%) | 0.65 | 0.29 | 0.06 | 0.08 | -0.98 | 0.44 | 0.22 | 0.64 | 1.14 |
| qR (%) | 6.35 | 3.19 | 0.67 | 1.75 | 4.01 | 0.50 | 2.44 | 5.96 | 16.63 |
| pH | 5.18 | 0.55 | 0.12 | 0.25 | -0.46 | 0.11 | 4.29 | 5.25 | 6.39 |
| ECe (µS/cm) | 232.2 | 126 | 26.27 | 1.08 | 0.31 | 0.54 | 93.00 | 206.00 | 538.0 |
| Total N (%) | 0.08 | 0.03 | 0.01 | -0.04 | -1.03 | 0.30 | 0.04 | 0.08 | 0.13 |
| SOC (%) | 0.65 | 0.29 | 0.06 | 0.08 | -0.98 | 0.44 | 0.22 | 0.64 | 1.14 |
| AC (mg/kg) | 322.2 | 137.44 | 28.66 | -0.04 | -0.92 | 0.43 | 70.69 | 315.27 | 554.1 |
| NPI | 0.96 | 0.23 | 0.05 | 1.34 | 2.57 | 0.24 | 0.68 | 0.88 | 1.68 |
| CPI | 0.70 | 0.20 | 0.04 | -0.44 | -0.79 | 0.29 | 0.32 | 0.74 | 0.98 |
| CL | 0.05 | 0.02 | 0.00 | 0.16 | -0.55 | 0.29 | 0.03 | 0.05 | 0.09 |
| Cli | 1.43 | 0.43 | 0.09 | 0.21 | -0.45 | 0.30 | 0.80 | 1.41 | 2.32 |
| CMI | 0.98 | 0.35 | 0.07 | -0.21 | -0.73 | 0.36 | 0.29 | 1.06 | 1.58 |
| nCMI | 62.22 | 22.12 | 4.61 | -0.21 | -0.73 | 0.36 | 18.42 | 67.03 | 100.2 |
| pb (g/cm^3^) | 1.88 | 0.16 | 0.03 | 0.48 | -0.33 | 0.09 | 1.65 | 1.83 | 2.26 |
| MaAS (%) | 44.68 | 21.08 | 4.39 | 0.02 | -1.58 | 0.47 | 13.98 | 45.53 | 73.76 |
| MiAS (%) | 14.50 | 5.85 | 1.22 | 0.48 | -1.13 | 0.40 | 7.04 | 13.52 | 25.54 |
| AS (%) | 59.17 | 17.15 | 3.58 | -0.31 | -0.95 | 0.29 | 24.13 | 59.74 | 81.49 |
| SI | 4.09 | 3.16 | 0.66 | 0.72 | -0.86 | 0.77 | 0.83 | 2.94 | 10.26 |
| PI | 10.18 | 9.03 | 1.88 | 0.45 | -1.54 | 0.89 | 0.85 | 3.69 | 26.71 |
| MWD (mm) | 0.75 | 0.61 | 0.13 | 0.49 | -1.48 | 0.81 | 0.13 | 0.35 | 1.85 |
| GMD (mm) | 0.51 | 0.20 | 0.04 | 0.42 | -1.04 | 0.40 | 0.25 | 0.54 | 0.92 |

SMB: soil microbial biomass; NBC: non-microbial biomass carbon; qR: microbial biomass carbon over total organic carbon; ECe: electric conductivity of soil; TN: total nitrogen; SOC: Soil organic carbon; AC: active carbon; NPI: nitrogen pool index; CPI: carbon pool index; CL: carbon lability; Cli: carbon lability index; CMI: carbon management index; nCMI: normalized carbon management index; pb: soil bulk density; MaAS: macroaggregate stability; MiAS: microaggregate stability; AS: total aggregate stability; SI: stability index; and PI: persistent index, MWD: Mean weight diameter; GMD: Geometric mean diameter. The control treatment is defined as conventional soybean–corn rotation under no-till management, with no gypsum application (0 Mg/ha) and no cover crop.
