# Supplementary material for: Minimum dataset with integrated scoring and indexing methods for soil quality assessment
Source: PLoS One. 2026 Apr 7;21(4):e0346136. doi: 10.1371/journal.pone.0346136 (PMC13056203; doi:10.1371/journal.pone.0346136)
Supplement: S6 Table — (DOCX) [file pone.0346136.s006.docx]

**S6 Table.** Load matrix and norm values of soil quality indicators evaluation for Indiana site (reference site).

| Soil properties | Principal component | | | | Norm value |
| --- | --- | --- | --- | --- | --- |
|  | PC1 | PC2 | PC3 | PC4 |  |
| SMB | 0.03 | **0.35** | -0.08 | **0.39** | 0.71 |
| Non-SMB | **0.30** | 0.06 | -0.13 | -0.02 | 0.99 |
| qR | -0.15 | 0.27 | 0.03 | 0.37 | 0.72 |
| pH | -0.06 | -0.22 | -0.03 | 0.02 | 0.47 |
| ECe | -0.05 | 0.10 | 0.09 | 0.14 | 0.29 |
| Total N | **0.29** | 0.10 | -0.07 | -0.01 | 0.96 |
| SOC | **0.30** | 0.07 | -0.13 | -0.01 | 0.99 |
| AC | 0.26 | 0.26 | -0.04 | -0.13 | 0.96 |
| NPI | **0.28** | -0.03 | -0.05 | -0.08 | 0.91 |
| CPI | **0.29** | -0.03 | -0.14 | -0.06 | 0.97 |
| CL | -0.14 | **0.34** | 0.24 | -0.27 | 0.92 |
| Cli | -0.14 | **0.34** | 0.24 | -0.27 | 0.92 |
| CMI | 0.26 | 0.20 | -0.02 | -0.23 | 0.92 |
| nCMI | 0.26 | 0.20 | -0.02 | -0.23 | 0.92 |
| pb | -0.10 | 0.04 | 0.13 | -0.09 | 0.40 |
| MaAS | 0.17 | 0.08 | 0.31 | 0.32 | 0.78 |
| MiAS | -0.10 | 0.24 | -0.33 | 0.09 | 0.81 |
| AS | 0.11 | 0.27 | 0.10 | **0.43** | 0.67 |
| SI | 0.10 | -0.19 | 0.33 | -0.02 | 0.76 |
| PI | 0.18 | -0.03 | **0.38** | 0.18 | 0.86 |
| MWD | 0.20 | -0.12 | **0.35** | 0.05 | 0.92 |
| GMD | 0.20 | -0.18 | **0.35** | -0.01 | 0.94 |
| Eigen value | 10.15 | 3.85 | 2.96 | 1.95 |  |
| Variance (%) | 42.3% | 16.0% | 12.3% | 8.1% |  |
| Cumulative Variance (%) | 42.3% | 58.3% | 70.7% | 78.8% |  |

Selected soil quality indicators for MDS_PCA_: SMB, Non-SMB, AS, and PI.

SMB: soil microbial biomass; Non-SMB: non-microbial biomass carbon; qR: microbial biomass carbon over total organic carbon; ECe: electric conductivity of soil; TN: total nitrogen; TC: Soil organic carbon; AC: active carbon; NPI: nitrogen pool index; CPI: carbon pool index; CL: carbon lability; Cli: carbon lability index; CMI: carbon management index; nCMI: normalized carbon management index; pb: soil bulk density; MaAS: macroaggregate stability; MiAS: microaggregate stability; AS: total aggregate stability; SI: stability index; and PI: persistent index, MWD: Mean weight diameter; GMD: Geometric mean diameter.
