# Supplementary material for: Minimum dataset with integrated scoring and indexing methods for soil quality assessment
Source: PLoS One. 2026 Apr 7;21(4):e0346136. doi: 10.1371/journal.pone.0346136 (PMC13056203; doi:10.1371/journal.pone.0346136)
Supplement: S6(a) Table — (DOCX) [file pone.0346136.s007.docx]

**S6(a) Table.** Pearson correlation coefficients among soil quality indicators and composite indices for the Indiana site (average of four replications, 2012–2016).

|  | SMB | Non-SMB | qR | pH | ECe | Total N | SOC | AC | NPI | CPI | CL | Cli | CMI | nCMI | pb | MaAS | MiAS | AS | SI | PI | MWD | GMD |
| --- | --- | --- | --- | --- | --- | --- | --- | --- | --- | --- | --- | --- | --- | --- | --- | --- | --- | --- | --- | --- | --- | --- |
| SMB | 1.00 | 0.18 | 0.76 | -0.19 | 0.18 | 0.24 | 0.22 | 0.35 | 0.04 | 0.07 | 0.15 | **0.15*** | 0.22 | 0.22 | 0.02 | 0.27 | 0.33 | 0.54 | -0.21 | 0.03 | -0.16 | -0.25 |
| Non-SMB |  | 1.00 | -0.43 | -0.20 | -0.15 | 0.95 | 1.00 | 0.88 | 0.84 | 0.94 | -0.44 | -0.44 | 0.85 | 0.85 | -0.35 | 0.40 | -0.15 | 0.35 | 0.15 | 0.40 | 0.47 | 0.43 |
| qR |  |  | 1.00 | -0.06 | 0.27 | -0.35 | -0.39 | -0.22 | -0.46 | -0.49 | 0.40 | 0.40 | -0.31 | -0.31 | 0.18 | -0.01 | 0.37 | 0.26 | -0.27 | -0.18 | -0.37 | -0.42 |
| pH |  |  |  | 1.00 | 0.05 | -0.26 | -0.20 | -0.30 | -0.15 | -0.11 | -0.19 | -0.19 | -0.25 | -0.25 | -0.01 | -0.24 | -0.06 | -0.31 | 0.05 | -0.07 | -0.01 | 0.06 |
| ECe |  |  |  |  | 1.00 | -0.15 | -0.14 | -0.09 | -0.14 | -0.14 | 0.17 | 0.17 | -0.07 | -0.07 | 0.10 | -0.02 | 0.10 | 0.06 | -0.12 | 0.06 | 0.01 | -0.03 |
| Total N |  |  |  |  |  | 1.00 | 0.95 | 0.88 | 0.90 | 0.86 | -0.34 | -0.34 | 0.83 | 0.83 | -0.33 | 0.48 | -0.17 | 0.41 | 0.24 | 0.42 | 0.47 | 0.42 |
| SOC |  |  |  |  |  |  | 1.00 | **0.89*** | 0.84 | 0.94 | -0.43 | -0.43 | 0.85 | 0.85 | -0.35 | 0.41 | -0.13 | 0.37 | 0.14 | 0.39 | 0.46 | 0.42 |
| AC |  |  |  |  |  |  |  | 1.00 | 0.68 | 0.74 | 0.01 | 0.01 | 0.93 | 0.93 | -0.24 | 0.41 | -0.03 | 0.44 | 0.06 | 0.34 | 0.35 | 0.28 |
| NPI |  |  |  |  |  |  |  |  | 1.00 | 0.92 | -0.46 | -0.46 | 0.80 | 0.80 | -0.24 | 0.38 | -0.32 | 0.19 | 0.36 | 0.38 | 0.51 | 0.52 |
| CPI |  |  |  |  |  |  |  |  |  | 1.00 | -0.55 | -0.55 | 0.84 | 0.84 | -0.28 | 0.32 | -0.23 | 0.19 | 0.21 | 0.35 | 0.49 | 0.49 |
| CL |  |  |  |  |  |  |  |  |  |  | 1.00 | 1.00 | -0.03 | -0.03 | 0.25 | -0.09 | 0.20 | 0.05 | -0.17 | -0.12 | -0.23 | -0.27 |
| Cli |  |  |  |  |  |  |  |  |  |  |  | 1.00 | -0.03 | -0.03 | 0.25 | -0.09 | 0.20 | 0.05 | -0.17 | -0.12 | -0.23 | -0.27 |
| CMI |  |  |  |  |  |  |  |  |  |  |  |  | 1.00 | 1.00 | -0.15 | 0.35 | -0.14 | 0.29 | 0.13 | 0.33 | 0.41 | 0.37 |
| nCMI |  |  |  |  |  |  |  |  |  |  |  |  |  | 1.00 | -0.15 | 0.35 | -0.14 | 0.29 | 0.13 | 0.33 | 0.41 | 0.37 |
| pb |  |  |  |  |  |  |  |  |  |  |  |  |  |  | 1.00 | -0.10 | -0.02 | -0.13 | 0.05 | -0.13 | -0.14 | -0.13 |
| MaAS |  |  |  |  |  |  |  |  |  |  |  |  |  |  |  | 1.00 | -0.49 | 0.77 | 0.43 | 0.64 | 0.55 | 0.48 |
| MiAS |  |  |  |  |  |  |  |  |  |  |  |  |  |  |  |  | 1.00 | 0.20 | -0.70 | -0.37 | -0.53 | -0.64 |
| AS |  |  |  |  |  |  |  |  |  |  |  |  |  |  |  |  |  | 1.00 | -0.04 | 0.45 | 0.23 | 0.08 |
| SI |  |  |  |  |  |  |  |  |  |  |  |  |  |  |  |  |  |  | 1.00 | 0.42 | 0.47 | 0.59 |
| PI |  |  |  |  |  |  |  |  |  |  |  |  |  |  |  |  |  |  |  | 1.00 | 0.91 | 0.84 |
| MWD |  |  |  |  |  |  |  |  |  |  |  |  |  |  |  |  |  |  |  |  | 1.00 | 0.97 |
| GMD |  |  |  |  |  |  |  |  |  |  |  |  |  |  |  |  |  |  |  |  |  | 1.00 |

Bolded values with asterisks represent statistically significant correlations at p < 0.05 (*), and p < 0.01 (**). SMB: soil microbial biomass; Non-SMB: non-microbial biomass carbon; qR: microbial biomass carbon over total organic carbon; ECe: electric conductivity of soil; TN: total nitrogen; SOC: Soil organic carbon; AC: active carbon; NPI: nitrogen pool index; CPI: carbon pool index; CL: carbon lability; Cli: carbon lability index; CMI: carbon management index; nCMI: normalized carbon management index; pb: soil bulk density; MaAS: macroaggregate stability; MiAS: microaggregate stability; AS: total aggregate stability; SI: stability index; and PI: persistent index, MWD: Mean weight diameter; GMD: Geometric mean diameter.
