# Supplementary material for: Minimum dataset with integrated scoring and indexing methods for soil quality assessment
Source: PLoS One. 2026 Apr 7;21(4):e0346136. doi: 10.1371/journal.pone.0346136 (PMC13056203; doi:10.1371/journal.pone.0346136)
Supplement: S7 Table — (DOCX) [file pone.0346136.s008.docx]

**S7 Table.** Load matrix and norm values of soil quality indicators evaluation for Hoytville (Ohio) site.

| Soil properties | Principal component | | | |  | Norm value |
| --- | --- | --- | --- | --- | --- | --- |
|  | PC1 | PC2 | PC3 | PC4 | PC5 |  |
| SMB | 0.13 | 0.03 | -0.09 | **0.51** | 0.42 | 0.87 |
| Non-SMB | 0.20 | 0.34 | 0.01 | -0.05 | -0.02 | 0.97 |
| qR | 0.08 | -0.05 | -0.09 | **0.54** | **0.44** | 0.90 |
| pH | -0.01 | 0.08 | 0.17 | -0.35 | **0.48** | 0.44 |
| ECe | 0.12 | -0.09 | -0.02 | 0.30 | -0.43 | 0.60 |
| Total N | 0.20 | 0.32 | -0.01 | 0.02 | -0.09 | 0.96 |
| SOC | 0.21 | 0.33 | 0.01 | -0.01 | 0.01 | 0.97 |
| AC | **0.31** | -0.03 | 0.19 | -0.01 | 0.02 | 0.97 |
| NPI | 0.02 | **0.40** | 0.03 | 0.01 | -0.09 | 0.96 |
| CPI | 0.13 | **0.39** | 0.03 | -0.01 | 0.02 | 0.94 |
| CL | 0.25 | -0.23 | 0.22 | 0.00 | 0.01 | 0.98 |
| Cli | 0.21 | -0.25 | 0.31 | 0.00 | 0.01 | 0.98 |
| CMI | 0.27 | 0.00 | 0.31 | -0.01 | 0.03 | 0.98 |
| nCMI | 0.27 | 0.00 | 0.31 | -0.01 | 0.03 | 0.98 |
| pb | -0.03 | -0.08 | 0.13 | -0.38 | 0.28 | 0.65 |
| MaAS | 0.25 | -0.09 | -0.29 | -0.10 | 0.04 | 0.92 |
| MiAS | -0.15 | 0.06 | **0.38** | 0.18 | -0.21 | 0.96 |
| AS | 0.24 | -0.09 | -0.05 | 0.04 | -0.16 | 0.90 |
| SI | 0.18 | -0.08 | **-0.35** | -0.16 | 0.13 | 0.94 |
| PI | 0.25 | -0.10 | -0.14 | -0.12 | -0.10 | 0.91 |
| MWD | 0.27 | -0.11 | -0.21 | -0.05 | -0.13 | 0.96 |
| GMD | 0.25 | -0.10 | -0.31 | -0.11 | -0.02 | 0.98 |
| Eigen value | 9.38 | 5.30 | 2.69 | 2.20 | 1.29 |  |
| Variance (%) | 39.1% | 22.1% | 11.2% | 9.2% | 5.4% |  |
| Cumulative variance (%) | 39.1% | 61.1% | 72.4% | 81.5% | 86.9% |  |

Selected soil properties for MDS_PCA_: qR, pH, AC, NPI and MiAs.

SMB: soil microbial biomass; Non-SMB: non-microbial biomass carbon; qR: microbial biomass carbon over total organic carbon; ECe: electric conductivity of soil; TN: total nitrogen; SOC: Soil organic carbon; AC: active carbon; NPI: nitrogen pool index; CPI: carbon pool index; CL: carbon lability; Cli: carbon lability index; CMI: carbon management index; nCMI: normalized carbon management index; pb: soil bulk density; MaAS: macroaggregate stability; MiAS: microaggregate stability; AS: total aggregate stability; SI: stability index; and PI: persistent index, MWD: Mean weight diameter; GMD: Geometric mean diameter.
