# Supplementary material for: Minimum dataset with integrated scoring and indexing methods for soil quality assessment
Source: PLoS One. 2026 Apr 7;21(4):e0346136. doi: 10.1371/journal.pone.0346136 (PMC13056203; doi:10.1371/journal.pone.0346136)
Supplement: S8 Table — (DOCX) [file pone.0346136.s010.docx]

**S8 Table.** Load matrix and norm values of soil quality indicators evaluation for Alabama site.

| Soil properties | Principal component | | | |  | Norm value |
| --- | --- | --- | --- | --- | --- | --- |
|  | PC1 | PC2 | PC3 | PC4 | PC5 |  |
| SMB | 0.15 | 0.08 | 0.24 | -0.27 | 0.36 | 0.87 |
| Non-SMB | **0.35** | -0.06 | -0.04 | 0.04 | 0.07 | 0.97 |
| qR | -0.22 | 0.11 | 0.22 | -0.27 | 0.15 | 0.90 |
| pH | 0.00 | 0.15 | -0.01 | 0.03 | 0.25 | 0.44 |
| ECe | 0.15 | -0.01 | 0.15 | -0.18 | -0.16 | 0.60 |
| Total N | 0.23 | 0.07 | 0.17 | -0.21 | -0.48 | 0.96 |
| SOC | **0.35** | -0.06 | -0.04 | 0.04 | 0.08 | 0.97 |
| AC | 0.26 | -0.11 | 0.28 | 0.19 | 0.09 | 0.97 |
| NPI | 0.20 | 0.06 | 0.17 | -0.19 | **-0.57** | 0.96 |
| CPI | 0.30 | -0.13 | -0.13 | 0.17 | 0.02 | 0.94 |
| CL | -0.23 | -0.04 | **0.39** | 0.10 | -0.05 | 0.98 |
| Cli | -0.21 | -0.03 | **0.41** | 0.09 | -0.04 | 0.98 |
| CMI | 0.16 | -0.18 | 0.28 | **0.34** | 0.02 | 0.98 |
| nCMI | 0.16 | -0.18 | 0.28 | **0.34** | 0.02 | 0.98 |
| pb | -0.09 | -0.12 | -0.07 | **0.32** | 0.11 | 0.65 |
| MaAS | 0.12 | **0.37** | 0.07 | 0.02 | 0.13 | 0.92 |
| MiAS | 0.02 | **-0.37** | 0.08 | -0.28 | 0.11 | 0.96 |
| AS | 0.18 | -0.03 | 0.19 | **-0.34** | 0.30 | 0.90 |
| SI | 0.01 | **0.37** | -0.02 | 0.26 | -0.05 | 0.94 |
| PI | 0.14 | 0.35 | 0.12 | -0.01 | 0.12 | 0.91 |
| MWD | 0.14 | **0.37** | 0.12 | -0.02 | 0.11 | 0.96 |
| GMD | 0.02 | **0.40** | -0.01 | 0.21 | -0.09 | 0.98 |
| Eigen value | 7.48 | 5.11 | 3.59 | 2.53 | 1.23 |  |
| Variance (%) | 31.2% | 21.3% | 14.9% | 10.5% | 5.1% |  |
| Cumulative variance (%) | 31.2% | 52.5% | 67.4% | 77.9% | 83.1% |  |

Selected soil properties for MDS_PCA_: SOC, NPI, Cli, CMI, pb, AS and GMD.

SMB: soil microbial biomass; Non-SMB: non-microbial biomass carbon; qR: microbial biomass carbon over total organic carbon; ECe: electric conductivity of soil; TN: total nitrogen; SOC: Soil organic carbon; AC: active carbon; NPI: nitrogen pool index; CPI: carbon pool index; CL: carbon lability; Cli: carbon lability index; CMI: carbon management index; nCMI: normalized carbon management index; pb: soil bulk density; MaAS: macroaggregate stability; MiAS: microaggregate stability; AS: total aggregate stability; SI: stability index; and PI: persistent index, MWD: Mean weight diameter; GMD: Geometric mean diameter.
