# Supplementary material for: Minimum dataset with integrated scoring and indexing methods for soil quality assessment
Source: PLoS One. 2026 Apr 7;21(4):e0346136. doi: 10.1371/journal.pone.0346136 (PMC13056203; doi:10.1371/journal.pone.0346136)
Supplement: S9 Table — (DOCX) [file pone.0346136.s012.docx]

**S9 Table.** Load matrix and norm values of soil quality indicators evaluation for Piketon (Ohio) site.

| Soil properties | Principal component | | | | Norm value |
| --- | --- | --- | --- | --- | --- |
|  | PC1 | PC2 | PC3 | PC4 |  |
| SMB | **0.25** | -0.02 | 0.17 | 0.17 | 0.85 |
| Non-SMB | **0.26** | 0.02 | -0.15 | -0.13 | 0.88 |
| qR | -0.09 | -0.14 | 0.34 | -0.29 | 0.72 |
| pH | 0.04 | 0.15 | -0.20 | **0.63** | 0.51 |
| ECe | 0.18 | 0.06 | 0.14 | -0.35 | 0.64 |
| Total N | **0.25** | 0.01 | -0.17 | -0.21 | 0.85 |
| SOC | **0.26** | 0.02 | -0.15 | -0.13 | 0.88 |
| AC | 0.22 | 0.26 | 0.01 | -0.19 | 0.86 |
| NPI | 0.06 | 0.09 | **-0.46** | -0.03 | 0.83 |
| CPI | 0.22 | 0.09 | -0.34 | -0.01 | 0.93 |
| CL | -0.12 | 0.39 | 0.21 | -0.03 | 0.93 |
| Cli | -0.13 | 0.38 | 0.20 | -0.01 | 0.92 |
| CMI | 0.05 | **0.45** | -0.08 | -0.02 | 0.91 |
| nCMI | 0.05 | **0.45** | -0.08 | -0.02 | 0.91 |
| pb | -0.21 | 0.04 | -0.04 | 0.30 | 0.69 |
| MaAS | **0.25** | 0.01 | 0.19 | 0.12 | 0.86 |
| MiAS | -0.23 | 0.09 | -0.20 | -0.19 | 0.83 |
| AS | 0.24 | 0.04 | 0.18 | 0.09 | 0.83 |
| SI | 0.24 | 0.00 | 0.20 | 0.20 | 0.83 |
| PI | **0.27** | 0.01 | 0.15 | 0.05 | 0.88 |
| MWD | **0.26** | 0.01 | 0.16 | 0.08 | 0.88 |
| GMD | **0.25** | -0.02 | 0.17 | 0.17 | 0.85 |
| Eigen value | 12.73 | 4.51 | 2.74 | 1.23 |  |
| Variance (%) | 53.0% | 18.8% | 11.4% | 5.1% |  |
| Cumulative variance (%) | 53.0% | 71.8% | 83.2% | 88.3% |  |

Selected soil properties for MDS_PCA_: pH, NPI, CMI, nCMI, and PI.

SMB: soil microbial biomass; Non-SMB: non-microbial biomass carbon; qR: microbial biomass carbon over total organic carbon; ECe: electric conductivity of soil; TN: total nitrogen; SOC: Soil organic carbon; AC: active carbon; NPI: nitrogen pool index; CPI: carbon pool index; CL: carbon lability; Cli: carbon lability index; CMI: carbon management index; nCMI: normalized carbon management index; pb: soil bulk density; MaAS: macroaggregate stability; MiAS: microaggregate stability;: total aggregate stability; SI: stability index; and PI: persistent index, MWD: Mean weight diameter; GMD: Geometric mean diameter.
