# Supplementary material for: Minimum dataset with integrated scoring and indexing methods for soil quality assessment
Source: PLoS One. 2026 Apr 7;21(4):e0346136. doi: 10.1371/journal.pone.0346136 (PMC13056203; doi:10.1371/journal.pone.0346136)
Supplement: S9(a) Table — (DOCX) [file pone.0346136.s013.docx]

**S9(a) Table.** Pearson correlation coefficients among soil quality indicators and composite indices for the Piketon (Ohio) site (average of four replications, 2012–2016).

|  | SMB | Non-SMB | qR | pH | ECe | Total N | SOC | AC | NPI | CPI | CL | Cli | CMI | nCMI | pb | MaAS | MiAS | AS | SI | PI | MWD | GMD |
| --- | --- | --- | --- | --- | --- | --- | --- | --- | --- | --- | --- | --- | --- | --- | --- | --- | --- | --- | --- | --- | --- | --- |
| SMB | 1.00 | **0.73*** | -0.20 | 0.10 | 0.52 | **0.68**** | **0.73**** | 0.63 | 0.02 | 0.53 | -0.32 | **-0.37** | 0.08 | 0.08 | -0.63 | 0.83 | -0.87 | 0.77 | 0.92 | **0.95**** | **0.96**** | **0.99**** |
| Non-SMB |  | 1.00 | -0.42 | 0.17 | 0.59 | 0.96 | 1.00 | 0.80 | 0.32 | 0.88 | -0.44 | -0.49 | 0.23 | 0.23 | -0.74 | 0.74 | -0.65 | 0.72 | 0.67 | **0.82**** | 0.81 | 0.73 |
| qR |  |  | 1.00 | -0.43 | 0.03 | -0.34 | -0.42 | -0.36 | -0.36 | -0.60 | 0.08 | 0.09 | -0.39 | -0.39 | 0.17 | -0.12 | 0.11 | -0.12 | -0.12 | -0.22 | -0.21 | -0.20 |
| pH |  |  |  | 1.00 | -0.06 | 0.10 | 0.17 | 0.17 | 0.25 | 0.35 | 0.05 | 0.05 | 0.34 | 0.34 | 0.11 | 0.15 | -0.06 | 0.18 | 0.13 | 0.10 | 0.11 | 0.10 |
| ECe |  |  |  |  | 1.00 | 0.56 | 0.59 | 0.63 | -0.03 | 0.40 | -0.10 | -0.15 | 0.21 | 0.21 | -0.51 | 0.60 | -0.46 | 0.61 | 0.53 | 0.63 | 0.61 | 0.52 |
| Total N |  |  |  |  |  | 1.00 | 0.96 | 0.77 | 0.46 | 0.82 | -0.43 | -0.48 | 0.20 | 0.20 | -0.73 | 0.68 | -0.60 | 0.67 | 0.61 | 0.77 | 0.76 | 0.68 |
| SOC |  |  |  |  |  |  | 1.00 | 0.80 | 0.32 | 0.88 | -0.44 | -0.49 | 0.23 | 0.23 | -0.74 | 0.74 | -0.65 | 0.72 | 0.67 | 0.82 | 0.81 | 0.73 |
| AC |  |  |  |  |  |  |  | 1.00 | 0.20 | 0.69 | 0.14 | 0.07 | 0.66 | 0.66 | -0.62 | 0.68 | -0.51 | 0.71 | 0.60 | 0.75 | 0.73 | 0.63 |
| NPI |  |  |  |  |  |  |  |  | 1.00 | 0.63 | -0.19 | -0.18 | 0.35 | 0.35 | -0.04 | -0.04 | 0.10 | -0.02 | 0.04 | 0.01 | 0.01 | 0.02 |
| CPI |  |  |  |  |  |  |  |  |  | 1.00 | -0.38 | -0.40 | 0.42 | 0.42 | -0.50 | 0.51 | -0.43 | 0.51 | 0.50 | 0.58 | 0.57 | 0.53 |
| CL |  |  |  |  |  |  |  |  |  |  | 1.00 | 1.00 | 0.66 | 0.66 | 0.33 | -0.26 | 0.40 | -0.20 | -0.25 | -0.29 | -0.29 | -0.32 |
| Cli |  |  |  |  |  |  |  |  |  |  |  | 1.00 | 0.64 | 0.64 | 0.38 | -0.32 | 0.45 | -0.26 | -0.30 | -0.35 | -0.35 | -0.37 |
| CMI |  |  |  |  |  |  |  |  |  |  |  |  | 1.00 | 1.00 | -0.03 | 0.13 | 0.08 | 0.20 | 0.13 | 0.14 | 0.14 | 0.08 |
| nCMI |  |  |  |  |  |  |  |  |  |  |  |  |  | 1.00 | -0.03 | 0.13 | 0.08 | 0.20 | 0.13 | 0.14 | 0.14 | 0.08 |
| pb |  |  |  |  |  |  |  |  |  |  |  |  |  |  | 1.00 | -0.63 | 0.60 | -0.61 | -0.56 | -0.71 | -0.69 | -0.63 |
| MaAS |  |  |  |  |  |  |  |  |  |  |  |  |  |  |  | 1.00 | -0.88 | 0.99 | 0.88 | 0.91 | 0.92 | 0.83 |
| MiAS |  |  |  |  |  |  |  |  |  |  |  |  |  |  |  |  | 1.00 | -0.79 | -0.86 | -0.84 | -0.84 | -0.87 |
| AS |  |  |  |  |  |  |  |  |  |  |  |  |  |  |  |  |  | 1.00 | 0.84 | 0.88 | 0.90 | 0.77 |
| SI |  |  |  |  |  |  |  |  |  |  |  |  |  |  |  |  |  |  | 1.00 | 0.87 | 0.89 | 0.92 |
| PI |  |  |  |  |  |  |  |  |  |  |  |  |  |  |  |  |  |  |  | 1.00 | 1.00 | 0.95 |
| MWD |  |  |  |  |  |  |  |  |  |  |  |  |  |  |  |  |  |  |  |  | 1.00 | 0.96 |
| GMD |  |  |  |  |  |  |  |  |  |  |  |  |  |  |  |  |  |  |  |  |  | 1.00 |

Bolded values with asterisks represent statistically significant correlations at p < 0.05 (*), and p < 0.01 (**).SMB: soil microbial biomass; Non-SMB: non-microbial biomass carbon; qR: microbial biomass carbon over total organic carbon; ECe: electric conductivity of soil; TN: total nitrogen; SOC: Soil organic carbon; AC: active carbon; NPI: nitrogen pool index; CPI: carbon pool index; CL: carbon lability; Cli: carbon lability index; CMI: carbon management index; nCMI: normalized carbon management index; pb: soil bulk density; MaAS: macroaggregate stability; MiAS: microaggregate stability;: total aggregate stability; SI: stability index; and PI: persistent index, MWD: Mean weight diameter; GMD: Geometric mean diameter.
