# Supplementary material for: Minimum dataset with integrated scoring and indexing methods for soil quality assessment
Source: PLoS One. 2026 Apr 7;21(4):e0346136. doi: 10.1371/journal.pone.0346136 (PMC13056203; doi:10.1371/journal.pone.0346136)
Supplement: S10 Table — (DOCX) [file pone.0346136.s014.docx]

**S10 Table.** Soil quality threshold values for Hoytville site (Ohio).

| Soil properties | Lower threshold (LT)  (score: 0%) | Critical or base threshold (CT or BT) (score: 50%) | Upper threshold (UT)  (score: 100%) | Optimum threshold (OT)  (score:100%) | Scoring  curve |
| --- | --- | --- | --- | --- | --- |
| SMB (mg/kg) | 100 | 310 | 514 |  | More is better |
| Non-SMB (%) | 1.5 | 1.87 | 2.23 |  | More is better |
| qR (%) | 0.7 | 1.6 | 2.7 |  | More is better |
| pH | 5.8 |  | 7.6 | 6.73 | Optimum |
| ECe (µS/cm) | 53 | 299 | 700 |  | Less is better |
| TN (%) | 0.173 | 0.198 | 0.225 |  | More is better |
| SOC (%) | 1.6 | 1.9 | 2.26 |  | More is better |
| AC (mg/kg) | 240 | 460 | 650 |  | More is better |
| NPI | 0.88 | 0.98 | 1.09 |  | More is better |
| CPI | 0.86 | 1.02 | 1.2 |  | More is better |
| CL | 0.01 | 0.02 | 0.03 |  | More is better |
| Cli | 0.72 | 1.22 | 1.63 |  | More is better |
| CMI | 0.7 | 1.22 | 1.65 |  | More is better |
| nCMI | 35 | 63 | 85 |  | More is better |
| Pb (g/cm^3^) | 1.22 | 1.37 | 1.53 |  | Less is better |
| MaAS (%) | 46 | 53.6 | 63 |  | More is better |
| MiAS (%) | 6.5 | 11.57 | 15 |  | Less is better |
| AS (%) | 58 | 65.27 | 71 |  | More is better |
| Sl | 3.0 | 4.72 | 7.5 |  | More is better |
| PI | 12 | 18.53 | 28 |  | More is better |
| MWD (mm) | 0.8 | 1.22 | 1.65 |  | More is better |
| GMD (mm) | 0.65 | 0.90 | 1.12 |  | More is better |

SMB: soil microbial biomass; Non-SMB: non-microbial biomass carbon; qR: microbial biomass carbon over total organic carbon; ECe: electric conductivity of soil; TN: total nitrogen; SOC: Soil organic carbon; AC: active carbon; NPI: nitrogen pool index; CPI: carbon pool index; CL: carbon lability; Cli: carbon lability index; CMI: carbon management index; nCMI: normalized carbon management index; pb: soil bulk density; MaAS: macroaggregate stability; MiAS: microaggregate stability; AS: total aggregate stability; SI: stability index; and PI: persistent index, MWD: Mean weight diameter; GMD: Geometric mean diameter.
