# Supplementary material for: Minimum dataset with integrated scoring and indexing methods for soil quality assessment
Source: PLoS One. 2026 Apr 7;21(4):e0346136. doi: 10.1371/journal.pone.0346136 (PMC13056203; doi:10.1371/journal.pone.0346136)
Supplement: S11 Table — (DOCX) [file pone.0346136.s015.docx]

**Table. S11.** Soil quality threshold values for Alabama site

| Soil  properties | Lower threshold (LT)  (score: 0%) | Critical or base threshold (CT or BT) (score: 50%) | Upper threshold (UT)  (score: 100%) | Optimum threshold (OT)  (score:100%) | Scoring  curve |
| --- | --- | --- | --- | --- | --- |
| SMB (mg/kg) | 8 | 82 | 200 |  | More is better |
| Non-SNB (%) | 0.34 | 0.7 | 1.1 |  | More is better |
| qR (%) | 0.3 | 1.18 | 2.32 |  | More is better |
| pH | 4.7 |  | 6.8 | 5.8 | Optimum |
| ECe (µS/cm) | 21 | 44 | 66 |  | Less is better |
| TN (%) | 0.02 | 0.053 | 0.09 |  | More is better |
| SOC (%) | 0.35 | 0.70 | 1.15 |  | More is better |
| AC (mg/kg) | 10 | 187 | 320 |  | More is better |
| NPI | 0.9 | 1.68 | 2.8 |  | More is better |
| CPI | 1.05 | 1.5 | 2.0 |  | More is better |
| CL | 0.00 | 0.03 | 0.06 |  | More is better |
| Cli | 0.04 | 0.73 | 1.35 |  | More is better |
| CMI | 0.1 | 1.09 | 2.0 |  | More is better |
| nCMI | 4.0 | 58.0 | 110 |  | More is better |
| Pb (g/cm^3^) | 1.5 | 1.82 | 2.1 |  | Less is better |
| MaAS (%) | 27 | 38 | 50 |  | More is better |
| MiAS (%) | 6 | 17.5 | 26 |  | Less is better |
| AS (%) | 46 | 55.29 | 64 |  | More is better |
| Sl | 0.5 | 2.33 | 5.0 |  | More is better |
| PI | 1 | 10 | 23 |  | More is better |
| MWD (mm) | 0.17 | 0.33 | 0.50 |  | More is better |
| GMD (mm) | 0.37 | 0.53 | 0.73 |  | More is better |

SMB: soil microbial biomass; Non-SMB: non-microbial biomass carbon; qR: microbial biomass carbon over total organic carbon; ECe: electric conductivity of soil; TN: total nitrogen; SOC: Soil organic carbon; AC: active carbon; NPI: nitrogen pool index; CPI: carbon pool index; CL: carbon lability; Cli: carbon lability index; CMI: carbon management index; nCMI: normalized carbon management index; pb: soil bulk density; MaAS: macroaggregate stability; MiAS: microaggregate stability; AS: total aggregate stability; SI: stability index; and PI: persistent index, MWD: Mean weight diameter; GMD: Geometric mean diameter.
