# Supplementary material for: Minimum dataset with integrated scoring and indexing methods for soil quality assessment
Source: PLoS One. 2026 Apr 7;21(4):e0346136. doi: 10.1371/journal.pone.0346136 (PMC13056203; doi:10.1371/journal.pone.0346136)
Supplement: S12 Table — (DOCX) [file pone.0346136.s016.docx]

**S12 Table.** Soil quality threshold values for Piketon (Ohio) site

| Soil properties | Lower threshold (LT)  (score: 0%) | Critical or base threshold (CT or BT) (score: 50%) | Upper threshold (UT)  (score: 100%) | Optimum threshold (OT)  (score:100%) | Scoring  curve |
| --- | --- | --- | --- | --- | --- |
| SMB (mg/kg) | 40 | 380 | 903 |  | More is better |
| Non-SMB (%) | 0.2 | 0.66 | 1.15 |  | More is better |
| qR (%) | 1.5 | 5.5 | 10.5 |  | More is better |
| pH | 4.8 |  | 6.7 | 5.8 | Optimum |
| ECe (µS/cm) | 25 | 254 | 534 |  | Less is better |
| TN (%) | 0.04 | 0.086 | 0.14 |  | More is better |
| SOC (%) | 0.2 | 0.70 | 1.24 |  | More is better |
| AC (mg/kg) | 150 | 378 | 630 |  | More is better |
| NPI | 0.6 | 0.97 | 1.4 |  | More is better |
| CPI | 0.35 | 0.75 | 1.15 |  | More is better |
| CL | 0.03 | 0.06 | 0.09 |  | More is better |
| Cli | 0.9 | 1.63 | 2.5 |  | More is better |
| CMI | 0.7 | 1.17 | 1.7 |  | More is better |
| nCMI | 40 | 74 | 110 |  | More is better |
| Pb (g/cm^3^) | 1.4 | 1.88 | 2.4 |  | Less is better |
| MaAS (%) | 5 | 47 | 90 |  | More is better |
| MiAS (%) | 3 | 16 | 26 |  | Less is better |
| AS (%) | 32 | 62 | 95 |  | More is better |
| Sl | 0 | 4 | 11 |  | More is better |
| PI | 0 | 11 | 32 |  | More is better |
| MWD (mm) | 0 | 0.81 | 2.1 |  | More is better |
| GMD (mm) | 0.1 | 0.48 | 1.0 |  | More is better |

SMB: soil microbial biomass; Non-SMB: non-microbial biomass carbon; qR: microbial biomass carbon over total organic carbon; ECe: electric conductivity of soil; TN: total nitrogen; SOC: Soil organic carbon; AC: active carbon; NPI: nitrogen pool index; CPI: carbon pool index; CL: carbon lability; Cli: carbon lability index; CMI: carbon management index; nCMI: normalized carbon management index; pb: soil bulk density; MaAS: macroaggregate stability; MiAS: microaggregate stability; AS: total aggregate stability; SI: stability index; and PI: persistent index, MWD: Mean weight diameter; GMD: Geometric mean diameter.
