# Supplementary material for: Minimum dataset with integrated scoring and indexing methods for soil quality assessment
Source: PLoS One. 2026 Apr 7;21(4):e0346136. doi: 10.1371/journal.pone.0346136 (PMC13056203; doi:10.1371/journal.pone.0346136)
Supplement: S13 Table — Calculated SQIw value was 0.59. (DOCX) [file pone.0346136.s017.docx]

**S13 Table.** Calculation of the soil quality index using weighted additive method (SQIw) (LSM1-no threshold) and total dataset (TDS) for the Indiana site (reference site). Calculated SQIw value was 0.59.

|  |  |  |  | |  |  |  |  |
| --- | --- | --- | --- | --- | --- | --- | --- | --- |
| Soil function | Weight | Soil  indicator | Sub-weight (A) | Scaled score | A XB | ∑(A XB) | C XW | SQI |
|  | W |  | A | B |  | C |  |  |
|  |  | Pb | 0.2 | 0.84 | 0.17 |  |  |  |
|  |  | MaAS | 0.2 | 0.88 | 0.18 |  |  |  |
| RDC | 0.4 | MiAS | 0.2 | 0.43 | 0.09 | 0.60 | 0.24 |  |
|  |  | MWD | 0.2 | 0.40 | 0.08 |  |  |  |
|  |  | GMD | 0.2 | 0.46 | 0.09 |  |  |  |
|  |  | CPI | 0.16 | 0.61 | 0.10 |  |  |  |
|  |  | CMI | 0.17 | 0.69 | 0.12 |  |  |  |
|  |  | AS | 0.17 | 0.90 | 0.15 |  |  |  |
| WSC | 0.2 | SI | 0.16 | 0.16 | 0.03 | 0.57 | 0.11 |  |
|  |  | PI | 0.17 | 0.49 | 0.08 |  |  |  |
|  |  | SOC | 0.17 | 0.55 | 0.09 |  |  | 0.59 |
|  |  | SMB | 0.09 | 0.35 | 0.03 |  |  |  |
|  |  | NBC | 0.08 | 0.51 | 0.04 |  |  |  |
|  |  | qR | 0.09 | 0.55 | 0.05 |  |  |  |
|  |  | pH | 0.08 | 0.83 | 0.07 |  |  |  |
|  |  | ECe | 0.08 | 0.46 | 0.04 |  |  |  |
|  |  | TN | 0.09 | 0.61 | 0.05 |  |  |  |
| NSC | 0.4 | AC | 0.09 | 0.62 | 0.06 | 0.58 | 0.23 |  |
|  |  | NPI | 0.08 | 0.63 | 0.05 |  |  |  |
|  |  | CPI | 0.08 | 0.61 | 0.05 |  |  |  |
|  |  | CL | 0.08 | 0.54 | 0.04 |  |  |  |
|  |  | Cli | 0.08 | 0.54 | 0.04 |  |  |  |
|  |  | nCMI | 0.08 | 0.69 | 0.05 |  |  |  |

SMB: soil microbial biomass; Non-SMB: non-microbial biomass carbon; qR: microbial biomass carbon over total organic carbon; ECe: electric conductivity of soil; TN: total nitrogen; SOC: Soil organic carbon; AC: active carbon; NPI: nitrogen pool index; CPI: carbon pool index; CL: carbon lability; Cli: carbon lability index; CMI: carbon management index; nCMI: normalized carbon management index; pb: soil bulk density; MaAS: macroaggregate stability; MiAS: microaggregate stability; AS: total aggregate stability; SI: stability index; and PI: persistent index, MWD: Mean weight diameter; GMD: Geometric mean diameter. RDC: Root development capacity; WSC: Water storage capacity; NSC: Nutrient storage capacity
