# Supplementary material for: Minimum dataset with integrated scoring and indexing methods for soil quality assessment
Source: PLoS One. 2026 Apr 7;21(4):e0346136. doi: 10.1371/journal.pone.0346136 (PMC13056203; doi:10.1371/journal.pone.0346136)
Supplement: S14 Table — The calculated SQIw value was 0.50. (DOCX) [file pone.0346136.s018.docx]

**S14 Table.** Calculation of the soil quality index using weighted additive method (SQIw) (LSM1-no threshold) and minimum dataset selection based on correlation with crop yield (MDS_Corr_) for Indiana site. The calculated SQIw value was 0.50.

| Soil function | Weight | Soil  indicator | Sub-weight (A) | Scaled score | A XB | ∑(A XB) | C XW | SQI |  |
| --- | --- | --- | --- | --- | --- | --- | --- | --- | --- |
|  | W |  | A | B |  | C |  |  |  |
|  |  | Pb | 0.2 | 0.50 | 0.10 |  |  |  |  |
|  |  | MaAS | 0.2 | 0.52 | 0.10 |  |  |  |  |
| RDC | 0.4 | MiAS | 0.2 | 0.51 | 0.10 | 0.50 | 0.20 |  |  |
|  |  | MWD | 0.2 | 0.47 | 0.09 |  |  |  |  |
|  |  | GMD | 0.2 | 0.49 | 0.10 |  |  |  |  |
| WSC | 0.2 | SOC | 1 | 0.48 | 0.48 | 0.48 | 0.10 | 0.50 |  |
|  |  | Non-SMB | 0.3 | 0.52 | 0.16 |  |  |  |  |
|  |  | TN | 0.3 | 0.49 | 0.15 |  |  |  |  |
| NSC | 0.4 | AC | 0.4 | 0.50 | 0.20 | 0.50 | 0.20 |  |  |

Non-SMB: non-microbial biomass carbon; TN: total nitrogen; SOC: Soil organic carbon; AC: active carbon; pb: soil bulk density; MaAS: macroaggregate stability; MiAS: microaggregate stability; MWD: Mean weight diameter; GMD: Geometric mean diameter. RDC: Root development capacity; WSC: Water storage capacity; NSC: Nutrient storage capacity
