# Supplementary material for: Minimum dataset with integrated scoring and indexing methods for soil quality assessment
Source: PLoS One. 2026 Apr 7;21(4):e0346136. doi: 10.1371/journal.pone.0346136 (PMC13056203; doi:10.1371/journal.pone.0346136)
Supplement: Attachment — Submitted filename: Response to reviewers comments.docx [file pone.0346136.s020.docx]

**Minimum Dataset with Integrated Scoring and Indexing Methods for Soil Quality Assessment**

**Manuscript Number: PONE-D-25-51945**

**Reviewer #1**

**Comments:**

This submission contributes to SQ literature by integrating quantitative approaches. As contextual, SQ shows to be site specific. It is adequately reflective and compelling, and well written. Improvements as follows:

Inferring soil compaction from bulk density as noted by “increasing pb (compaction)” is likely unfound given there are contrasting regional sites where inherent attributes such as texture can be driving pb, instead of management. Soils with different pb can arrive and show the same detrimental compaction, and vice versa, different pb can result in the same yield outcome. For instance, sandy soils can have relatively high bulk density with exhibiting compaction as they are well aerated and pores are interconnected. As pb is not a functional variable, but mostly descriptive, its usefulness for the purpose of informing compaction in a SQ study is questionable or at least debatable due to introducing confounding influence.

**Response:**

We thank the reviewer for this insightful and constructive comment, which helped improve the conceptual clarity and interpretation of bulk density within the soil quality (SQ) framework. We agree that bulk density (pb) is a descriptive physical property rather than a direct functional indicator of soil compaction, and that its interpretation can be confounded by inherent soil attributes such as texture, pore connectivity, and mineralogy, particularly across contrasting regional sites.

In response, we have revised the manuscript to remove any direct inference of soil compaction from increasing pb values. Specifically, the phrasing equating “increasing pb” with “compaction” has been eliminated. Instead, bulk density is now interpreted as a supporting structural descriptor, reflecting soil packing conditions that may influence crop productivity depending on site-specific context.

As shown on **page 8**, we explicitly state that bulk density alone does not uniquely diagnose soil compaction, particularly across soils with contrasting textures, and we clarify that soils with similar pb values may differ substantially in pore connectivity, aeration, and root growth resistance. Accordingly, pb is treated within this study as a descriptive physical indicator of soil structure, rather than a direct functional measure of compaction, thereby reducing the potential for confounding interpretation.

These revisions ensure that the role of bulk density in the SQ assessment is conceptually sound, scientifically defensible, and consistent with current soil physics understanding, while preserving its value as part of a multivariate soil quality framework.

**Comments:**

Soil depth increments are presented in inconsistent way through the paper. Please cross check the document and improve this expression consistently.

**Response:**

We thank the reviewer for identifying this issue. We agree that consistent expression of soil depth increments is essential for clarity and accurate interpretation of soil physical, chemical, and biological indicators.

In response, we have systematically revised the manuscript to standardize soil depth terminology throughout the document. Specifically: **In the Abstract**, soil depth is now consistently expressed as the 0–30 cm soil profile, reflecting the combined depth used in the soil quality index (SQI) analysis.

**In the Materials and Methods**, Data source section (**page 6**), we clearly state that soil samples were collected at two fixed depth increments (0–15 and 15–30 cm) and subsequently averaged to represent the 0–30 cm soil profile for SQI calculations.

Ambiguous or inconsistent phrasing (e.g., use of “vs.” or mixed depth notation) has been removed to avoid confusion and to ensure consistency across sections.

**Comments:**

For informing introduction and discussion sections, recent work on a wider range of cropping systems elsewhere has shown non-linear weighted additive indexing approach to outperform other options in terms of their sensitivity and effectiveness to arrive at SQ indexes – can this be reconciled in the text?. For example, as relevant for this aspect, refer and check: Iheshiulo et al. 2024. Quantitative evaluation of soil health based on a minimum dataset under various short-term crop rotations on the Canadian prairies. Science of The Total Environment 935, 173335. In particular, the discussion section shows lack of references to put the results into a broader context of the available literature.

**Response:**

We thank the reviewer for this valuable suggestion and for highlighting recent advances in soil quality indexing approaches. **We agree that recent studies, including Iheshiulo et al. (2024)**, have demonstrated improved sensitivity of nonlinear scoring combined with weighted additive indexing under certain cropping systems.

In response, we have revised both the Introduction and the Discussion sections to explicitly acknowledge and reconcile these findings with our results.

In the Introduction, **we now reference recent evidence** showing that nonlinear weighted additive indexing can enhance sensitivity in some agroecosystems and frame our study as a systematic comparison and cross-site evaluation of linear and nonlinear scoring functions combined with additive, weighted additive, and Nemoro indexing approaches.

In the Discussion, we explicitly compare our findings with those of **Iheshiulo et al. (2024)** and clarify that while nonlinear weighted additive indexing may outperform other approaches in specific systems (e.g., short-term crop rotations in the Canadian prairies), our results indicate that linear scoring combined with additive or weighted additive indexing produced more consistent and transferable SQI values across diverse soils and regions in the United States.

We further emphasize that these differences likely arise from variations in soil types, climatic conditions, cropping systems, indicator selection strategies, and the incorporation of crop yield as a deductive criterion for minimum dataset development. Collectively, these revisions clarify that the performance of scoring and indexing methods is context dependent and that no single scoring–indexing combination is universally optimal, thereby reconciling our findings with recent literature.

Introduction:

Discussion:

Introduction

**Comments:**

In particular, the discussion section shows lack of references to put the results into a broader context of the available literature.

**Response:**

In response to the reviewer’s comment, we strengthened the Discussion by explicitly linking key findings to relevant soil quality literature at multiple points, including variability among scoring and indexing methods, linear versus nonlinear scoring behavior, dataset selection approaches, sensitivity analysis, and threshold normalization.

**Comments:**

“non-SMB” is puzzling in the submission. Although Table 3 indicates a statistically significant positive Pearson correlation with grain yield, Table 4 states “Less is better” for this variable and derivate. One would anticipate that having more carbon in non SMB pool (like in about any pool in soils) would result in improving ecosystem functions, including maize productivity and hence showing an upper asymptote while reaching saturation. In fact, non-SMB-C pretty much resembles SOC or TC data, which in Table 4 shows as “more is better”. Please elaborate, correct or address because this is concerning given that non-SMB was selected as part of MDS.

**Response:**

Thank you for this important and insightful comment. We agree that the description of non-SMB and its associated scoring direction required clarification.

Non-SMB carbon represents soil organic carbon (SOC) after subtracting the microbial biomass carbon (SMB) fraction. Because SMB constitutes only a small but dynamic portion of SOC, non-SMB is numerically very similar to SOC (Non-SMB=SOC-SMB(C); SOC=SMB+Non-SMB; Non-SMB≈SOC since SMB<<<SOC) and is therefore strongly autocorrelated with it. Conceptually, however, non-SMB does not represent an inert carbon pool; it includes both relatively stable and protected labile fractions such as particulate organic matter and permanganate-oxidizable carbon.

We acknowledge that the designation of “Less is better” in Table 4 was inconsistent with the positive Pearson correlation observed with grain yield (Table 3) and with established understanding that greater soil organic carbon generally enhances soil functions and productivity. This inconsistency has now been corrected. The scoring direction has been revised to align with the observed positive yield relationship and ecological interpretation.

Furthermore, given the strong overlap and autocorrelation between SOC and non-SMB, retaining both variables in the MDS could introduce redundancy and multicollinearity. To improve conceptual clarity and avoid duplication, we have retained SOC as the representative carbon indicator and removed non-SMB from the final MDSCorr framework. This modification does not alter the overall interpretation of soil carbon dynamics or the performance of the optimized SQI model.

The manuscript, Table 4, and related text have been revised accordingly.

**Comments:**

It feels disjointed and even redundant to read a paragraph starting as “In conclusion, the analysis” just before the section entitled "Conclusions”. Also initial sentences of the "Conclusions” section reads more as a narrative summary of the study while it would read better to directly state the key findings or lessons learned.

**Response:**

We thank the reviewer for this helpful comment. In response, we have removed the phrase “In conclusion” from the final paragraph of the Discussion to eliminate redundancy with the Conclusions section and improve narrative flow.

Additionally, we have revised the opening of the Conclusions section to directly state the key findings and lessons learned, rather than providing a narrative summary of the study. The revised Conclusions now emphasize the main outcomes, including the sensitivity of soil quality indices to methodological choices and the superior performance of the MDSCorr framework combined with linear scoring and additive indexing.These revisions improve clarity, cohesion between sections, and readability of the manuscript.

**Comments:**

Right panel of Fig. 6 for non linear scoring method simply shows two clusters right-top corner, and left-bottom corner, and there are no data points in the intermediate range. From below 0.485 to above 0.495 in the horizontal x axis, this sizable open gap in the data makes unsubstantiate the provided robust linear regressions due to uncertainty. The presented R2, as high as 93%, are an artifact of having just two extreme clusters, drawing unsupported linear regressions in between, and nothing in between the clusters of data points to inform the goodness of fitness over the entire range, and enable definite statements in the text of the abstract and discussion section.

**Response:**

We thank the reviewer for this insightful observation and agree that the clustered distribution of data points in the nonlinear scoring panel of Fig. 6 warrants cautious interpretation. In response, we have revised the manuscript to qualify the interpretation of regression results under nonlinear scoring.

Specifically, we now clarify that nonlinear scoring methods compress SQI variability into a narrow range, which results in clustered data and limited mid-range coverage. As a result, the high R² values observed under nonlinear scoring primarily reflect agreement between clustered endpoint values rather than robust linear relationships across the full SQI range. We have revised the Discussion text accordingly to avoid over-interpretation and to emphasize that linear scoring provides a more informative assessment of agreement between MDS_Corr_ and TDS due to its broader SQI variability.

These revisions ensure that the statistical interpretation of Fig. 6 is accurate, transparent, and appropriately qualified.

**Reviewer #2:**

I went through the paper, and I found that generally, the topic is interesting. However, it needs major revisions before being accepted for publication. The comments are listed below:

**Comments:**

Abstract: It is better to present those properties that were recognized as crucial factors in the calculation of SQI in the studied region.

**Response:**

Thank you for this valuable suggestion. We have revised the Abstract to explicitly identify the key soil properties recognized as most influential in SQI calculation for the studied regions. Specifically, we now state that the crop yield–correlated minimum dataset (MDSCorr) is driven by a reduced set of indicators representing soil nutrient status (e.g., nitrogen and organic carbon pools), biologically active carbon, and aggregate-related physical properties associated with soil structure and stability. This revision improves clarity and ensures that the Abstract more clearly communicates the practical and scientific relevance of the identified indicators without duplicating detailed results presented in the main text.

**Comments:**

Page 6 – Paragraph 1: Provide more details on the total number of soil data, and also the number of soil data studied in each site separately.

**Response:**

We appreciate this suggestion. The manuscript has been revised to explicitly report the total number of soil samples analyzed and the number of samples collected at each site. Specifically, we now state that 576 geo-referenced composite soil samples were included in the analysis, with 144 samples from each of the four study sites

**Comments:**

Page 8 – Total dataset: First, the presentation of the properties is not appropriate and should be revised, particularly in terms of the use of parentheses and abbreviations to ensure clarity. Second, there are 11 chemical properties, not 12 — please correct this. In addition, specify that MWD and GMD are related to aggregate size distribution or primary particle size distribution.

**Response:**

Thank you for pointing this out. We revised the presentation of the total dataset to improve clarity and consistency in the use of abbreviations and parentheses. The total number of indicators was corrected to 21, comprising 11 chemical, 8 physical, and 2 biological properties. In addition, mean weight diameter (MWD) and geometric mean diameter (GMD) are now explicitly described as indicators of soil aggregate size distribution.

**Comments:**

Page 8 – Selection of minimum dataset ...: Some of the properties selected for the MDS may be correlated with each other. For instance, MWD and GMD might show a strong and significant correlation. Therefore, including properties with correlation coefficients lower than 0.5 in relation to corn yield may lead to improved results. The Variance Inflation Factor (VIF) can be a useful indicator of multicollinearity among soil properties. It is recommended to select variables with correlation coefficients greater than ± 0.4 and retain only one of the two properties when their VIF exceeds 5. This approach could enhance the reliability of the analysis and potentially yield better results than the current method. At least, it would be worthwhile to test this procedure.

**Response:**

We appreciate this insightful suggestion. To address potential multicollinearity among the selected MDSCorr indicators, we evaluated variance inflation factors (VIF) for all retained variables. The results indicated that VIF values were below the commonly accepted threshold (VIF < 5), suggesting that multicollinearity was not sufficiently strong to require exclusion of any indicators. Although some aggregation-related variables (e.g., MWD and GMD) were moderately correlated, they were retained because they represent complementary aspects of soil aggregate size distribution and respond differently to management and soil texture. A clarification regarding this assessment has been added to the manuscript

**Comments:**

Page 11: All equations should be numbered consecutively in the main text.

**Response:**

Thank you for the comment. All equations in the main text have now been renumbered consecutively using Arabic numerals. The Nash efficiency coefficient and relative deviation coefficient equations have been revised and are now presented as Eqs. (3) and (4) in the revised manuscript.

**Comments:**

Page 13 – The last paragraph: Blue line and red line of which figure? Address the related figure.

**Response:**

Thank you for this helpful comment. We agree that the previous wording referring to “blue lines” and “red lines” was ambiguous and did not clearly identify the corresponding figure elements.

To address this, we revised the text to remove all color-based references and explicitly referred to the relevant figure panels. The paragraph has been rewritten to describe the results using Fig. 3(a) (without threshold normalization) and Fig. 3(b) (with threshold normalization), clearly distinguishing between linear and nonlinear scoring methods, dataset types (TDS, MDSCorr, MDSPCA), and indexing approaches (SQIa and SQIw).

**Comments:**

Table 5: Define the abbreviations and symbols in the footnote of the table.

**Response:**

Thank you for this helpful suggestion. We have revised Table 5 by adding a comprehensive footnote that defines all abbreviations and symbols used in the table, including dataset types (TDS, MDS_Cor_r, MDS_PCA_), scoring methods (LN, NLN), and indexing methods (SQIₐ, SQI_w_, SQIₙ). This clarification improves the readability and interpretability of the table for readers.

**Comments:**

Page 15 – The last paragraph: Correlation normally shows with r, not r2. Check this index. In addition, r² is always positive. Why was it calculated as r2 = - 0.52? Check and revise these statements.

R2 is coefficient of determination which tells us how much variability in Y is accounted by X.

**Response:**

Thank you for this important clarification. We agree that correlations should be reported using Pearson’s *r*, whereas R² represents the coefficient of determination and is always non-negative. We have revised the text to report correlation coefficients (*r*) when discussing relationships among scoring methods and retained R² only when describing variance explained by linear regression. The previously reported negative “R²” value has been corrected accordingly. These revisions improve both the statistical accuracy and clarity of interpretation.

**Comments:**

Page 16: correct (R2 = 0.27 0.7)

**Response:**

Thank you for noting the formatting issue. The reported coefficients of determination have been corrected and are now consistently presented as ranges where appropriate (e.g., R² = 0.27–0.70 for MDSPCA). The text and Fig. 6 have been revised to ensure consistency and to clarify that high R² values under nonlinear scoring reflect clustered agreement rather than predictive performance across the full SQI range.

**Comments:**

Table 6: correct “sensityvity” in the title

**Response:**

Thank you for pointing this out. The spelling of “sensityvity” has been corrected to “sensitivity” in the title of Table 6.

**Comments:**

Page 18: Regarding the statement “The positive Ef values across all locations …”. Just being positive Ef values does not show a very good accuracy and superiority of a relationship. Ef value varies from - ∞ to 1. A proper relationship should have an Ef value more than 0.65 and not just positive. So, I suggest revising this sentence.

**Response:**

Thank you for this important clarification. We agree that positive Ef values alone do not necessarily indicate good model performance. The text has been revised to explicitly state that Ef values exceeding commonly accepted thresholds (E_f_ ≥ 0.65) indicate good to excellent agreement. In the revised manuscript, we clarify that MDS_Corr_-based SQIs consistently achieved Ef values between 0.65 and 0.94 across all sites, whereas MDS_PCA_-based SQIs exhibited low or negative Ef values, indicating inferior performance.

**Comments:**

Page 19: No need to define the Ef and ER again. Once defined in the main text is enough.

**Response:**

Thank you for this helpful suggestion. We have removed the repeated definitions of the Nash–Sutcliffe efficiency coefficient (Ef) and the relative deviation coefficient (ER) from Page 179. These metrics are now only referenced without redefinition, as they are already clearly defined earlier in the Methods section.

**Comments:**

Page 19: No need to mention “Supporting Tables S6-S9”. Just write as Tables S6-S9.

**Response:**

We appreciate this editorial comment. The text has been revised accordingly, and all references to “Supporting Tables S6–S9” have been corrected to “Tables S6–S9” to maintain consistency with journal formatting guidelines.

**Comments:**

Discussion section: Overall, the Discussion section requires improvement. The authors have primarily focused on the methods used for developing the SQI; however, it is also essential to discuss the key soil parameters identified and their relationships with soil quality in the studied field and cropping system. This is a critical aspect of the paper, and the Discussion section should be strengthened accordingly.

**Response:**

We thank the reviewer for this important comment. The Discussion section has been substantially strengthened by adding a new subsection that interprets the key soil quality indicators identified by the MDSCorr approach. This section now explicitly discusses the roles of dominant chemical and physical soil properties and their relationships with soil functioning and corn-based cropping systems, thereby improving the agronomic and ecological interpretation of the results.

**Comments:**

Conclusion: Provide more specific suggestions for future works and studies.

**Response:**

Thank you for this helpful suggestion. The Conclusions section has been revised to improve clarity and conciseness and to explicitly include specific directions for future research. In particular, we now highlight the need for (i) validating the MDSCorr framework across a broader range of soil types, cropping systems, and climatic conditions; (ii) incorporating additional biological indicators to enhance sensitivity to management-induced soil changes; (iii) refining region-specific threshold values using long-term datasets; and (iv) integrating the proposed SQI framework with digital soil mapping and decision-support tools. These additions strengthen the practical implications and forward-looking relevance of the study.

**Editorial Comments and Responses**

**Comments:**

1. Please ensure that your manuscript meets PLOS ONE's style requirements, including those for file naming. The PLOS ONE style templates can be found at

[https://journals.plos.org/plosone/s/file?id=wjVg/PLOSOne_formatting_sample_main_body.pdf](https://urldefense.com/v3/__https:/journals.plos.org/plosone/s/file?id=wjVg*PLOSOne_formatting_sample_main_body.pdf__;Lw!!KGKeukY!2Xt8clrKUI_OchrhyYjJ41lwEg2Qj2J_ZT1Z9fTVkzqVRsfQ4qyH1I71MRA9BcswSTZBiaF8fTEcIbnH6Q$) and

[https://journals.plos.org/plosone/s/file?id=ba62/PLOSOne_formatting_sample_title_authors_affiliations.pdf](https://urldefense.com/v3/__https:/track.editorialmanager.com/CL0/https:*2F*2Fjournals.plos.org*2Fplosone*2Fs*2Ffile*3Fid=ba62*2FPLOSOne_formatting_sample_title_authors_affiliations.pdf/1/010f019b71720299-29d049f6-627d-45dc-8559-e7c06f7f068f-000000/uXWM8YPnArmyFkp-mv6W0SBO8GdmaUJQlT7UFZ63hdY=243__;JSUlJSUlJQ!!KGKeukY!2Xt8clrKUI_OchrhyYjJ41lwEg2Qj2J_ZT1Z9fTVkzqVRsfQ4qyH1I71MRA9BcswSTZBiaF8fTEiM9HaaQ$)

**Response:**

Thank you for this helpful suggestion. We have carefully reviewed the PLOS ONE style requirements using the provided templates and have revised our manuscript accordingly. The formatting and file naming have been updated to fully comply with the journal’s guidelines.

If you would like, I can also help refine responses to other editorial comments to ensure consistency and professionalism throughout your rebuttal letter.

**Comments:**

2. Thank you for stating the following in the Acknowledgments Section of your manuscript:

“United Soybean Board, Grant/Award Number: 1520-732-7226; The Ohio State University; USDA-ARS.”

We note that you have provided additional information within the Acknowledgements Section that is not currently declared in your Funding Statement. Please note that funding information should not appear in the Acknowledgments section or other areas of your manuscript. We will only publish funding information present in the Funding Statement section of the online submission form.

Please remove any funding-related text from the manuscript and let us know how you would like to update your Funding Statement. Currently, your Funding Statement reads as follows:

“United Soybean Board, Grant/Award Number: 1520-732-7226; The Ohio State University; USDA-ARS.”

Please include your amended statements within your cover letter; we will change the online submission form on your behalf.

**Response:** Thank you for this clarification. We have removed all funding-related information from the Acknowledgments section of the manuscript, as requested. The manuscript has been revised accordingly.

**Comments:**

3. Please include captions for your Supporting Information files at the end of your manuscript, and update any in-text citations to match accordingly. Please see our Supporting Information guidelines for more information: [http://journals.plos.org/plosone/s/supporting-information](https://urldefense.com/v3/__http:/track.editorialmanager.com/CL0/http:*2F*2Fjournals.plos.org*2Fplosone*2Fs*2Fsupporting-information/1/010f019b71720299-29d049f6-627d-45dc-8559-e7c06f7f068f-000000/KU43CcV7ik7BiaWtF9eqKbk_fAw-I6-B-Mm6WwuuAfY=243__;JSUlJSU!!KGKeukY!2Xt8clrKUI_OchrhyYjJ41lwEg2Qj2J_ZT1Z9fTVkzqVRsfQ4qyH1I71MRA9BcswSTZBiaF8fTEajI9Pmg$).

**Response:**

Thank you for this helpful guidance. We have added the captions for all Supporting Information files at the end of the manuscript, in accordance with the journal’s guidelines. Additionally, we have carefully reviewed and updated all in-text citations to ensure they correctly correspond to the Supporting Information files.

**Comments:**

4. We note that Figure 9 in your submission contain [map/satellite] images which may be copyrighted. All PLOS content is published under the Creative Commons Attribution License (CC BY 4.0), which means that the manuscript, images, and Supporting Information files will be freely available online, and any third party is permitted to access, download, copy, distribute, and use these materials in any way, even commercially, with proper attribution. For these reasons, we cannot publish previously copyrighted maps or satellite images created using proprietary data, such as Google software (Google Maps, Street View, and Earth). For more information, see our copyright guidelines: [http://journals.plos.org/plosone/s/licenses-and-copyright](https://urldefense.com/v3/__http:/journals.plos.org/plosone/s/licenses-and-copyright__;!!KGKeukY!2Xt8clrKUI_OchrhyYjJ41lwEg2Qj2J_ZT1Z9fTVkzqVRsfQ4qyH1I71MRA9BcswSTZBiaF8fTEHMnfPvg$).

We require you to either (1) present written permission from the copyright holder to publish these figures specifically under the CC BY 4.0 license, or (2) remove the figures from your submission:

 (1) You may seek permission from the original copyright holder of Figure 9 to publish the content specifically under the CC BY 4.0 license.

We recommend that you contact the original copyright holder with the Content Permission Form ([http://journals.plos.org/plosone/s/file?id=7c09/content-permission-form.pdf](https://urldefense.com/v3/__http:/journals.plos.org/plosone/s/file?id=7c09*content-permission-form.pdf__;Lw!!KGKeukY!2Xt8clrKUI_OchrhyYjJ41lwEg2Qj2J_ZT1Z9fTVkzqVRsfQ4qyH1I71MRA9BcswSTZBiaF8fTHKCN0mpg$)) and the following text:

“I request permission for the open-access journal PLOS ONE to publish XXX under the Creative Commons Attribution License (CCAL) CC BY 4.0 ([http://creativecommons.org/licenses/by/4.0/](https://urldefense.com/v3/__http:/creativecommons.org/licenses/by/4.0/__;!!KGKeukY!2Xt8clrKUI_OchrhyYjJ41lwEg2Qj2J_ZT1Z9fTVkzqVRsfQ4qyH1I71MRA9BcswSTZBiaF8fTEa0OcJqw$)). Please be aware that this license allows unrestricted use and distribution, even commercially, by third parties. Please reply and provide explicit written permission to publish XXX under a CC BY license and complete the attached form.”

Please upload the completed Content Permission Form or other proof of granted permissions as an "Other" file with your submission.

In the figure caption of the copyrighted figure, please include the following text: “Reprinted from [ref] under a CC BY license, with permission from [name of publisher], original copyright [original copyright year].”

 (2) If you are unable to obtain permission from the original copyright holder to publish these figures under the CC BY 4.0 license or if the copyright holder’s requirements are incompatible with the CC BY 4.0 license, please either i) remove the figure or ii) supply a replacement figure that complies with the CC BY 4.0 license. Please check copyright information on all replacement figures and update the figure caption with source information. If applicable, please specify in the figure caption text when a figure is similar but not identical to the original image and is therefore for illustrative purposes only.

The following resources for replacing copyrighted map figures may be helpful:

USGS National Map Viewer (public domain): [http://viewer.nationalmap.gov/viewer/](https://urldefense.com/v3/__http:/track.editorialmanager.com/CL0/http:*2F*2Fviewer.nationalmap.gov*2Fviewer*2F/1/010f019b71720299-29d049f6-627d-45dc-8559-e7c06f7f068f-000000/wlEF5QIErKssX3-2rtHghKge5NPtlOL6zp3Q8my_LVE=243__;JSUlJQ!!KGKeukY!2Xt8clrKUI_OchrhyYjJ41lwEg2Qj2J_ZT1Z9fTVkzqVRsfQ4qyH1I71MRA9BcswSTZBiaF8fTGQQHZFPw$)

The Gateway to Astronaut Photography of Earth (public domain): [http://eol.jsc.nasa.gov/sseop/clickmap/](https://urldefense.com/v3/__http:/track.editorialmanager.com/CL0/http:*2F*2Feol.jsc.nasa.gov*2Fsseop*2Fclickmap*2F/1/010f019b71720299-29d049f6-627d-45dc-8559-e7c06f7f068f-000000/JkztzFHR1DjNxKdiEuiIoKU2bbiuEc3WmR8dlrYOj6Q=243__;JSUlJSU!!KGKeukY!2Xt8clrKUI_OchrhyYjJ41lwEg2Qj2J_ZT1Z9fTVkzqVRsfQ4qyH1I71MRA9BcswSTZBiaF8fTHc_-09vQ$)

Maps at the CIA (public domain): [https://www.cia.gov/library/publications/the-world-factbook/index.html](https://urldefense.com/v3/__https:/www.cia.gov/library/publications/the-world-factbook/index.html__;!!KGKeukY!2Xt8clrKUI_OchrhyYjJ41lwEg2Qj2J_ZT1Z9fTVkzqVRsfQ4qyH1I71MRA9BcswSTZBiaF8fTExz1844A$) and [https://www.cia.gov/library/publications/cia-maps-publications/index.html](https://urldefense.com/v3/__https:/track.editorialmanager.com/CL0/https:*2F*2Fwww.cia.gov*2Flibrary*2Fpublications*2Fcia-maps-publications*2Findex.html/1/010f019b71720299-29d049f6-627d-45dc-8559-e7c06f7f068f-000000/r72d8e5lDB4CcZM1gwDiY0-5ht6jIsYbJrvpRXR_lMo=243__;JSUlJSUl!!KGKeukY!2Xt8clrKUI_OchrhyYjJ41lwEg2Qj2J_ZT1Z9fTVkzqVRsfQ4qyH1I71MRA9BcswSTZBiaF8fTHQAjTGNQ$)

NASA Earth Observatory (public domain): [http://earthobservatory.nasa.gov/](https://urldefense.com/v3/__http:/track.editorialmanager.com/CL0/http:*2F*2Fearthobservatory.nasa.gov*2F/1/010f019b71720299-29d049f6-627d-45dc-8559-e7c06f7f068f-000000/696el0Kc9GkgWhUlKRWF_vc089OTYE-Qqq-kj32IEik=243__;JSUl!!KGKeukY!2Xt8clrKUI_OchrhyYjJ41lwEg2Qj2J_ZT1Z9fTVkzqVRsfQ4qyH1I71MRA9BcswSTZBiaF8fTHo9JUepg$)

Landsat: [http://landsat.visibleearth.nasa.gov/](https://urldefense.com/v3/__http:/track.editorialmanager.com/CL0/http:*2F*2Flandsat.visibleearth.nasa.gov*2F/1/010f019b71720299-29d049f6-627d-45dc-8559-e7c06f7f068f-000000/7bZ27QH7SK8Y3FvXZBcbAP2xhmbFc7HA6tEkqgeyoGU=243__;JSUl!!KGKeukY!2Xt8clrKUI_OchrhyYjJ41lwEg2Qj2J_ZT1Z9fTVkzqVRsfQ4qyH1I71MRA9BcswSTZBiaF8fTFVXOwM_A$)

USGS EROS (Earth Resources Observatory and Science (EROS) Center) (public domain): [http://eros.usgs.gov/#](https://urldefense.com/v3/__http:/track.editorialmanager.com/CL0/http:*2F*2Feros.usgs.gov*2F*23/1/010f019b71720299-29d049f6-627d-45dc-8559-e7c06f7f068f-000000/IQCFB83aDZKB8whfLFeOX5a6J5tt3dmGOz0ZaNW7XLs=243__;JSUlJQ!!KGKeukY!2Xt8clrKUI_OchrhyYjJ41lwEg2Qj2J_ZT1Z9fTVkzqVRsfQ4qyH1I71MRA9BcswSTZBiaF8fTGdeAzheg$)

Natural Earth (public domain): [http://www.naturalearthdata.com/](https://urldefense.com/v3/__http:/track.editorialmanager.com/CL0/http:*2F*2Fwww.naturalearthdata.com*2F/1/010f019b71720299-29d049f6-627d-45dc-8559-e7c06f7f068f-000000/gM4vPnFrtvSbUT7qJeEa-mZ04kGHfVSoOU0MZQc0TmQ=243__;JSUl!!KGKeukY!2Xt8clrKUI_OchrhyYjJ41lwEg2Qj2J_ZT1Z9fTVkzqVRsfQ4qyH1I71MRA9BcswSTZBiaF8fTEI4is6Kw$)

5. If the reviewer comments include a recommendation to cite specific previously published works, please review and evaluate these publications to determine whether they are relevant and should be cited. There is no requirement to cite these works unless the editor has indicated otherwise.

**Response:**

Thank you for bringing this to our attention. To comply with PLOS ONE’s copyright and licensing requirements, we have removed all maps and satellite images from the manuscript. **Figure 9** has been revised accordingly to eliminate any potential copyright conflicts. The updated figure now fully adheres to the journal’s CC BY 4.0 licensing policy.
